# Supplementary material for: Organic Nitrogen Supplementation Increases Vegetative and Reproductive Biomass in a Versatile White Rot Fungus
Source: J Fungi (Basel). 2022 Dec 20;9(1):7. doi: 10.3390/jof9010007 (PMC9865380; doi:10.3390/jof9010007)
Supplement: Supplementary file 1 [file jof-09-00007-s001.zip › jof-2028944-supplementary.pdf]

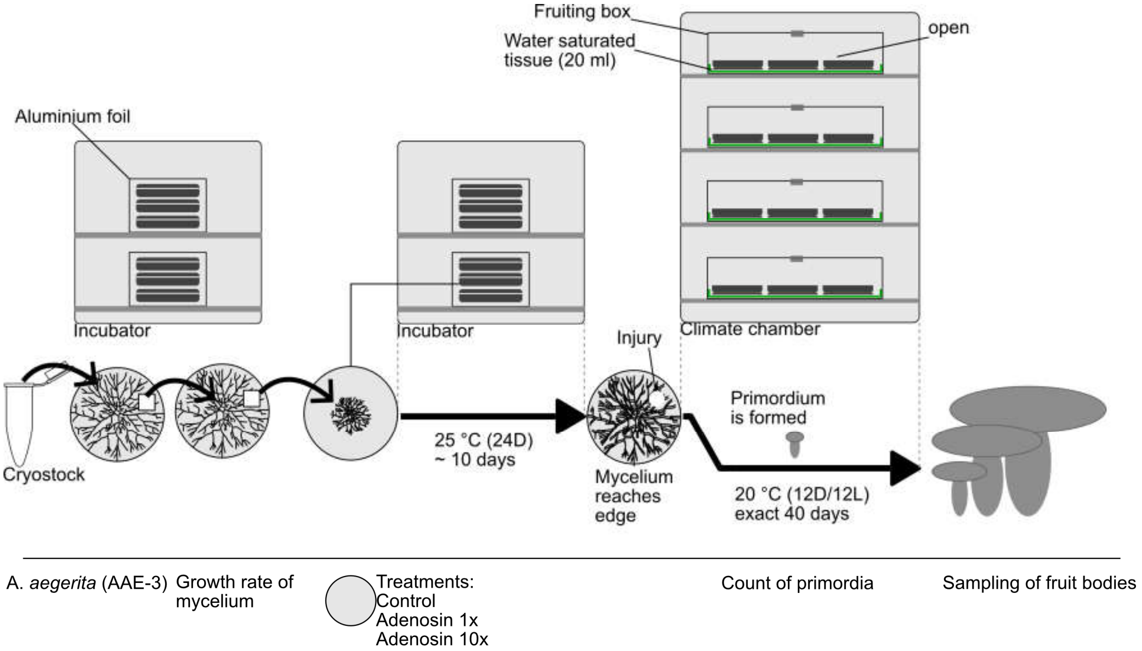

Figure S1. Illustration of experimental setup. Growth of mycelium in agar plates for growth rate assessment. After the colonization of the plates to the edge, fruiting induction took place chiefly by transfer from the incubator (WTB Binder, Tuttlingen, Germany, 24 h dark) to the climate chamber (12 h/12 h dark/light). Primordia were counted 3 times a week for 40 days. After 40 days, all fruiting bodies were harvested as reproductive biomass.

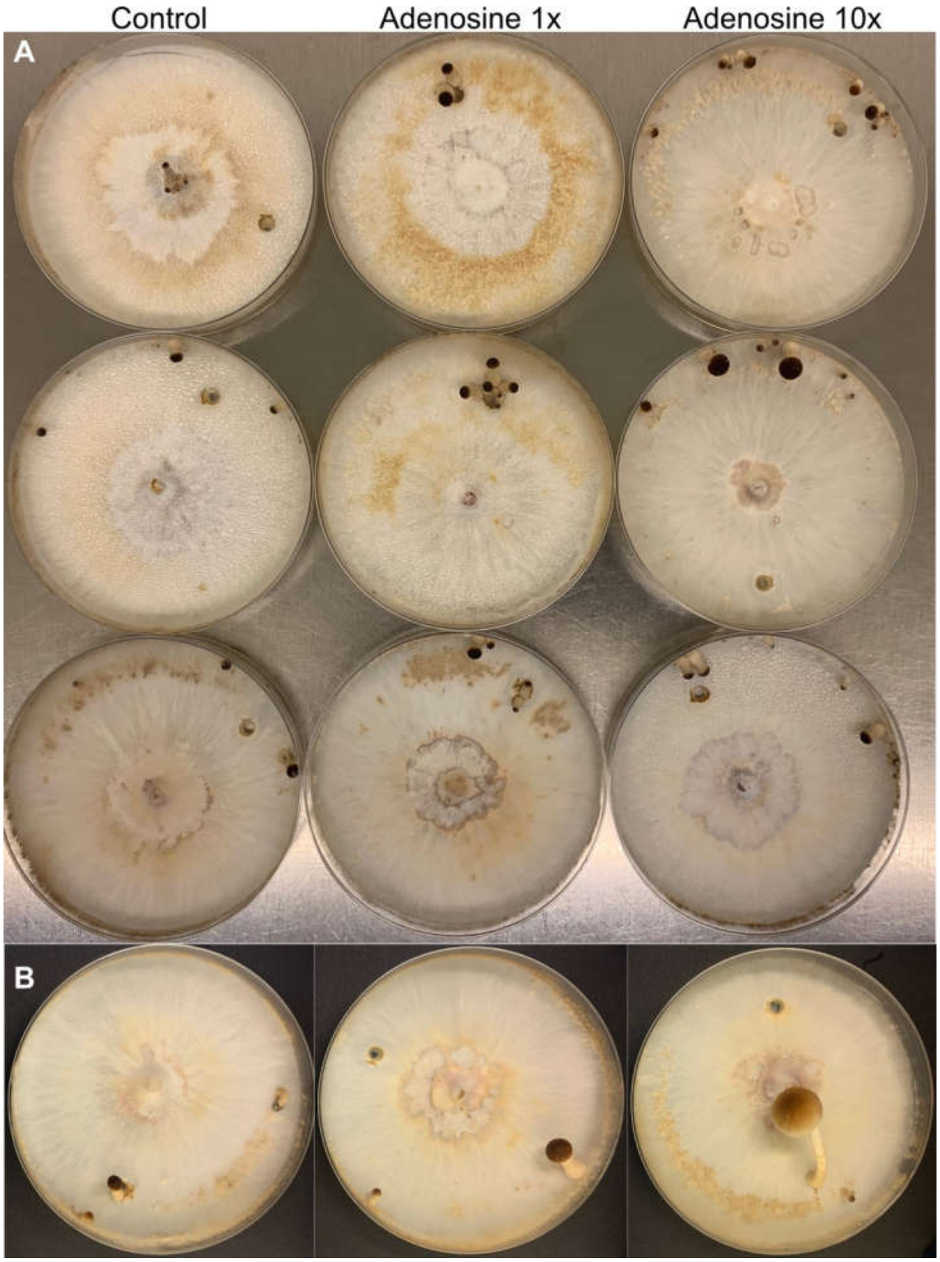

Figure S2. Reproductive biomass assessment A) Number of fruiting body primordia after 11 days of fruiting induction. A representative triplicate of plates is shown with each treatment. B) Reproductive biomass after 40 days of cultivation post-fruiting induction. A representative plate is shown for each treatment.
